# Clinical Profile of COVID-19 Patients Admitted at a Private Hospital During Three Surges in Mandalay, Myanmar

**DOI:** 10.7759/cureus.35167

**Published:** 2023-02-19

**Authors:** Kyu Kyu, Tin Ko Ko, Zin Mar Lwin, May Kyi Soe, Kyaw Win Maw, Aung Myo Thant, Kyi Shin, Moe Kyaw Myint

**Affiliations:** 1 Internal Medicine, City Hospital, Mandalay, MMR; 2 Surgery, City Hospital, Mandalay, MMR; 3 Hospital Medicine, City Hospital, Mandalay, MMR; 4 Health Systems Research, Department of Medical Research, Myanmar Health Ministry, Pyin Oo Lwin, MMR

**Keywords:** covid-19, private hospital, myanmar, waves, clinical profile

## Abstract

Introduction

During the coronavirus disease 2019 (COVID-19) pandemic, private hospitals in Mandalay started to manage COVID-19 infections according to national treatment guidelines since February 2021. Variations of clinical characteristics and their outcomes in different surges could be evaluated in the private hospital. This study aimed to assess the clinical profile and outcomes of COVID-19 patients admitted at a private hospital during three surges in Mandalay.

Methods

This study is a retrospective record review of the case series of COVID-19 patients admitted at City Hospital, Mandalay. The study was conducted from January to December 2022. All of the hospital records of COVID-19 patients admitted during the second wave from February 2020 to 26 May 2021, the third wave from 27 May 2021 to 27 January 2022, and the fourth wave from 28 January to April 2022 were included in the study.

Results

A total of 1606 admitted cases were included in the study. The mean with standard deviation (SD) of age was 55.7±18.5, and males were 778 (48.4%). The mean duration of hospital stay in days was 10.8±5.94, 10.6±6.11, and 7.3±2.88 in second, third, and fourth waves, respectively. The mean duration of hospital stay was shortened in the fourth wave. Comorbid conditions with hypertension and/or diabetes diseases were mostly observed in three waves of COVID-19 infection. Fever was the most presented symptom in three waves. Cough, sore throat, and rhinorrhea were observed more in the fourth wave compared with previous waves. Complication with pneumonia (71.3%), liver dysfunction (21.0%), acute respiratory distress syndrome (10.0%), thrombocytopenia (6.2%), acute kidney injury (5.5%), bleeding (3.9%), and pulmonary embolism (2.9%) were investigated. Antiviral treatment such as remdesivir or molnupiravir was used more in the patients of third and fourth waves than those of the second wave. Oxygen therapy (59.9%), prone position (35.5%), non-invasive ventilation (9.5%), invasive ventilation (0.5%), inotropes (4.6%), and renal replacement therapy (1.1%) were recorded in serious cases. Only 7.9% and 9.4% died in the hospital in second and third waves. No mortality was observed in the fourth wave.

Conclusions

The study recommended that COVID-19 patients with comorbid conditions of hypertension or diabetes and ages 65 and older should be taken with intensive care support at the hospital. This study also concluded that a private hospital in Mandalay could tackle with COVID-19 severe cases in line with national treatment guidelines since the second wave and could provide better management in the fourth wave. Antiviral treatment should be used in severe COVID-19 cases for further emergency management. In conclusion, private hospital involvement in the COVID-19 pandemic is supportive of the healthcare provision in Myanmar in an emergency situation.

## Introduction

The coronavirus disease 2019 (COVID-19) pandemic transmission started at Wuhan, China, in late 2019. It is an infectious disease caused by severe acute respiratory syndrome coronavirus 2 (SARS-CoV-2). COVID-19 patients may present with many symptoms such as fever, cough, anorexia, fatigue, shortness of breath, and myalgia. Moreover, flu-like symptoms are also presented. Clinical presentations may vary in accordance with regional variation, pandemic waves, and pathogen alteration. COVID-19 pandemic waves occur due to the rising of infection transmission with new variant appearances in countries [1]. Regarding the diagnosis, clinical presentation is highly supportive. Nucleic acid amplifying test of respiratory specimens such as reverse transcription-polymerase chain reaction (RT-PCR) for COVID-19 infection is used as laboratory confirmatory diagnosis. However, rapid antigen testing is useful if RT-PCR is not available in some conditions with limited resources [2]. In the World Health Organization’s (WHO) living guidance paper, other differential diagnoses such as influenza, dengue, malaria, typhoid, and respiratory tract infections are not negligible in hospital management [3].

Critical COVID-19 cases such as acute respiratory distress syndrome (ARDS) and septic shock are cared aligned with standard management guideline [4]. Treatment with antimicrobial therapy for co-infections and secondary infections coinciding with COVID-19 has to be started as soon as possible according to laboratory investigations, clinical judgment, local epidemiology, and patient host factor [5]. COVID-19 patients are manifested with neurological, neuropsychiatric, and mental problems. Those should be treated appropriately at the hospital [6]. If the disease becomes very severe, intubation with mechanical ventilation is a lifesaving management in advanced hospitals. Antiviral as a specific treatment for severe disease is also practicable. Remdesivir, a ribonucleic acid (RNA)-dependent polymerase inhibitor, is the most promising broad-spectrum antiviral agent that resulted from clinical studies [7,8].

Severe COVID-19 patients may be cured and die. The mortality of severe and critically ill patients may be different substantially in different case series all over the world. The World Health Organization (WHO) recommended collecting clinical data of admitted COVID-19 patients from countries all over the world and contributing clinical characterization data at the WHO Global COVID-19 Clinical Data Platform [1].

In Myanmar, the first case of COVID-19 infection was detected on 23 March 2020. Then, an outbreak of infection started, and new cases reached up to 124,630 in 2020 and 530,834 in 2021. The Ministry of Health (MoH) had response actions on the COVID-19 pandemic through the Coronavirus Disease 2019 (COVID-19) Containment and Emergency Response Committee. The MoH laid down the national management guidelines in line with local contexts. The Department of Public Health, Department of Medical Services, and Department of Medical Research conduct the preparedness and response strategic actions on COVID-19 transmission. In 2020, clinical treatment for hospitalized patients was focused by public hospitals such as secondary-level (township/district) hospitals and tertiary-level (state/regional/general) hospitals. However, because of increasing transmission and human resource shortage that resulted from an unstable country situation, the support from private sector involvement was needed to manage the surge of infection. Therefore, in early 2021, some specialized private hospitals were allowed to treat COVID-19 patients in line with national treatment guidelines [9].

During the COVID-19 pandemic in Myanmar, there were four surges called as waves: the first wave was from 23 March to 15 August 2020, the second wave from 16 August 2020 to 26 May 2021, the third wave from 27 May 2021 to 27 January 2022, and the fourth wave from 28 January 2022 up to the present. These waves were determined by the Central Epidemiological Unit of the Department of Public Health and approved by the Ministry of Health, Myanmar. Therefore, variations of clinical characteristics in different waves have been practiced in the private hospitals. These clinical characteristics and their outcomes among the patients are evaluated by waves. Therefore, this study aimed to assess the clinical profile and outcomes of COVID-19 patients admitted at a private hospital in Mandalay.

## Materials and methods

This study is a retrospective record review of the case series of COVID-19 patients admitted at a private, city hospital at Mandalay. It is 300-bedded specialist hospital equipped with high-technology medical care units, intensive care units, high-standard medical laboratories, and operation theaters. This study was conducted from January to December 2022. All of the hospital records of COVID-19 patients admitted at City Hospital from February 2021 to April 2022 were included in this study. During the COVID-19 pandemic in Myanmar, there were four waves: the first wave was from 23 March to 15 August 2020, the second wave from 16 August 2020 to 26 May 2021, the third wave from 27 May 2021 to 27 January 2022, and the fourth wave from 28 January 2022 up to the present. According to wave distribution, the studied cases were distributed in three waves: second, third, and fourth waves. The patients who presented to the hospital with no symptoms but needed investigation, the patients with clinical signs of pneumonia, and the suspected patients with comorbid conditions and severely ill cases were confirmed with either rapid diagnostic testing or PCR testing.

All of the COVID-19-confirmed cases were admitted to the hospital except the mild cases without risk or comorbid conditions. Those mild cases were transferred to the regional-level clinical management committee. Then, the admitted cases were categorized and treated according to national management guidelines for COVID-19 of Myanmar. The symptomatic patients without evidence of viral pneumonia or hypoxia were defined as mild disease; adolescents or adults with clinical signs of pneumonia (fever, cough, dyspnea, and fast breathing) as moderate disease (pneumonia); adolescents or adults with clinical signs of pneumonia (fever, cough, dyspnea, and fast breathing) plus either respiratory rate of >30 breaths/minute, severe respiratory distress, or saturation of peripheral oxygen (SpO_2_) of <93% on room air as severe disease (severe pneumonia); and complications such as ARDS, organ failure, sepsis, and shock as critically ill. This study included all COVID-19-confirmed cases. Hospital records of patients who had a history of COVID-19 infection and were admitted for the second time for another disease were not enrolled in this study. The patients who were vaccinated and re-admitted for COVID-19 infections were included in the study. Both the hospital patient chart and the electronic medical record (EMR) system of the hospital were used as a source of data document. The clinical data were collected in case record form (CRF) modified from the standard case record form (CRF) developed by the WHO Guideline Development Group for the WHO Global COVID-19 Clinical Data Platform [1].

Data analysis

The data on clinical characteristics, laboratory investigations, treatment, and final outcome status were reviewed using the electronic medical record (EMR). Individual clinical data were abstracted after discharge from hospital. The data on the clinical profile of COVID-19 patients were collected with CRF developed using the WHO Global COVID-19 Clinical Data Platform. Data entry and analysis were done using the Statistical Package for Social Sciences (SPSS) software (version 18.0, PASW Statistics, Chicago, IL). Descriptive analysis was carried out presenting with mean, medium, standard deviation (SD), and interquartile range (IQR) for continuous numerical data and frequency and percentage for categorical data. The variables among the waves were analyzed using chi-square (χ^2^) test for comparing categorical data, analysis of variance (ANOVA) for comparing means, and nonparametric test (median test) for comparing medians. Statistical significance was set at p-value of <0.05.

Ethical approval

The study was conducted after obtaining the ethical approval from Research Ethics Committee of University of Medicine, Mandalay (approval number: 1535/UMM/Research, dated 14 June 2022). The waiver of informed consent taking in conduct of this retrospective record review was approved by the Research Ethics Committee. This research was registered at Myanmar Health Research Registry (http://www.mhrr-mohs.com) (PLRID-00192_V14).

## Results

**Figure 1:**
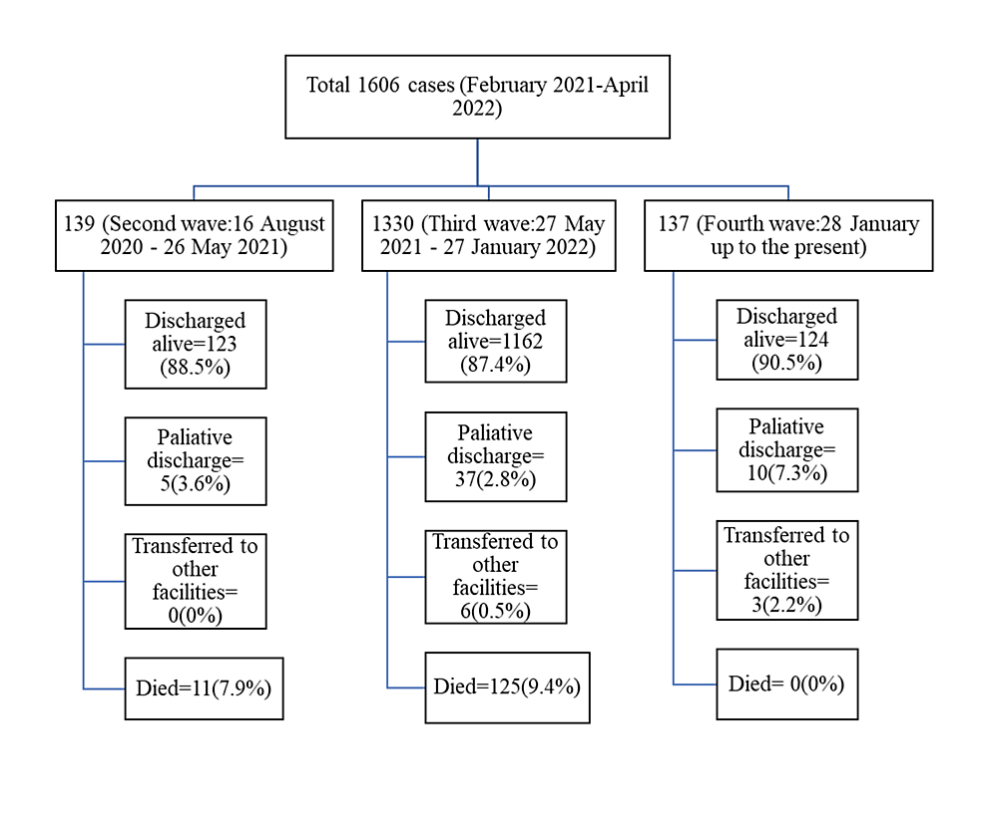
STROBE diagram showing the distribution of cases and their outcomes by waves. STROBE: Strengthening the Reporting of Observational Studies in Epidemiology

Between 8 February 2021 and 14 April 2022, a total of 1606 cases were admitted at City Hospital, Mandalay, for COVID-19 infections. Among them, 67 (4.2%) were re-admitted as post-COVID-19 complications, and 306 (19.1%) were vaccinated. The patients were distributed in the second, third, and fourth waves. A total of 139 cases in the second wave, 1330 cases in the third wave, and 137 cases in the fourth wave were reviewed. The following Strengthening the Reporting of Observational Studies in Epidemiology (STROBE) diagram shows the distribution of cases by waves and their outcomes (Figure 1).

In this study, the mean (±SD) age of a total of 1606 cases was 55.7±18.5. The mean age by wave distribution showed 58.2±16.8, 54.35±18.34, and 65.6±18.1 in the second, third, and fourth waves, respectively. More than half of them were within 51 and 75 years old age group. Males were 778 (48.4%), and females were 828 (51.6%). Among them, healthcare workers were 128 (8.0%), and laboratory workers were six (0.4%). Pregnant mothers were 24 (1.5%) (Table 1).

**Table 1:** Sociodemographic characteristics of the COVID-19 patients admitted at City Hospital by waves. SD, standard deviation; COVID-19, coronavirus disease 2019

Variables	Second wave (n=139)	Third wave (n=1330)	Fourth wave (n=137)	Total (n=1606)	P-value
Age (mean±SD)	58.2±16.8	54.35±18.34	65.6±18.1	55.7±18.5	<0.001
Age group					
<25 years	7 (5.0)	81 (6.1)	4 (2.9)	92 (5.7)	<0.001
25-50 years	29 (20.9)	420 (31.6)	18 (13.1)	467 (29.1)	
51-75 years	89 (64.0)	667 (50.2)	71 (51.8)	827 (51.5)	
>75 years	14 (10.1)	162 (12.2)	44 (32.1)	220 (13.7)	
Gender					
Male	79 (56.8)	629 (47.3)	70 (51.1)	778 (48.4)	0.082
Female	60 (43.2)	701 (52.7)	67 (48.9)	828 (51.6)	
Healthcare worker	12 (8.6)	114 (8.6)	2 (1.5)	128 (8.0)	0.013
Laboratory worker	0 (0)	5 (0.4)	1 (0.7)	6 (0.4)	0.495
Pregnant	1 (0.7)	21 (1.6)	2 (1.5)	24 (1.5)	0.682

Table 2 shows the baseline characteristics and comorbid conditions of the COVID-19 patients. Among the hospital patients, comorbid conditions with hypertension or diabetes diseases were mostly observed in three waves of COVID-19 infection in City Hospital, Mandalay, with 48.2%, 39.8%, and 54.0% for hypertension and 41.0%, 34.3%, and 39.4% for diabetes in the second, third, and fourth waves, respectively. The vaccination program in Myanmar had been initiated since January 2021, so almost all of the patients had not received vaccination in the second wave. Cumulatively, about 80.9% of the patients had not received any dose of vaccination. Among the 1606 cases, there were 67 (4.2%) cases that were re-admitted as post-COVID-19 complications.

**Table 2:** Baseline characteristics and comorbidity conditions of the COVID-19 patients on admission. SpO_2_, saturation of peripheral oxygen; ART, antiretroviral therapy; BPH, benign prostatic hypertrophy; ACEI, angiotensin-converting enzyme inhibitor; ARB, angiotensin receptor blocker; NSAID, nonsteroidal anti-inflammatory drug; SD, standard deviation; COVID-19, coronavirus disease 2019

Variables	Second wave (n=139)	Third wave (n=1330)	Fourth wave (n=137)	Total (n=1606)	P-value
Temperature (degree Fahrenheit) (mean±SD)	99.0±0.9	98.7±0.7	98.9±1.1	98.8±0.7	<0.001
Heart rate (beat per minute) (mean±SD)	92.9±16.3	93.6±17.1	93.5±21.4	93.6±17.4	0.900
Systolic blood pressure (mmHg) (mean±SD)	131.8±18.9	131.3±19.9	138.2±26.8	131.7±20.6	0.001
Diastolic blood pressure (mmHg) (mean±SD)	81.2±10.8	82.7±12.0	84.2±15.2	82.7±12.2	0.124
Respiratory rate (mean±SD)	23.4±4.9	23.3±10.1	22.8±7.5	23.3±9.6	0.857
SpO_2_ (%) (mean±SD)	92.4±9.4	93.7±7.7	95.9±5.2	93.80±7.7	0.001
O_2_ supply on admission					
Room air	97 (69.8)	986 (74.1)	108 (78.8)	1191 (74.2)	0.086
Oxygen therapy	42 (30.2)	344 (25.9)	29 (21.2)	415 (25.8)	
Comorbidity					
Chronic cardiac disorder	17 (12.2)	168 (12.6)	40 (29.2)	225 (14.0)	<0.001
Hypertension	67 (48.2)	529 (39.8)	74 (54.0)	670 (41.7)	<0.002
Chronic pulmonary disease	4 (2.9)	33 (2.5)	5 (3.6)	42 (2.6)	0.692
Asthma	6 (4.3)	28 (2.1)	7 (5.1)	41 (2.6)	0.041
Chronic kidney disease	8 (5.8)	78 (5.9)	19 (13.9)	105 (6.5)	0.007
Chronic liver disease	3 (2.2)	34 (2.6)	9 (6.6)	46 (2.9)	0.024
Chronic neurological disorder	5 (3.6)	43 (3.2)	20 (14.6)	68 (4.2)	<0.001
HIV on ART	0 (0)	7 (0.5)	0 (0)	7 (0.4)	0.409
HIV not on ART	0 (0)	2 (0.2)	1 (0.7)	3 (0.2)	
Diabetes	57 (41.0)	456 (34.3)	54 (39.4)	567 (35.3)	0.166
Current smoking	10 (7.2)	76 (5.7)	8 (5.8)	94 (5.9)	0.629
Tuberculosis	0 (0)	2 (0.2)	0 (0)	2 (0.1)	0.812
Malignancy	2 (1.4)	17 (1.3)	3 (2.2)	22 (1.4)	0.681
Anemia	11 (7.9)	95 (7.1)	10 (7.3)	116 (7.2)	0.945
Hypothyroid	4 (2.9)	28 (2.1)	3 (2.2)	35 (2.2)	0.838
Obesity	1 (0.7)	36 (2.7)	4 (2.9)	41 (2.6)	0.354
Prediabetes	1 (0.7)	42 (3.2)	6 (4.4)	49 (3.1)	0.077
Arthritis	6 (4.3)	42 (3.2)	5 (3.6)	53 (3.3)	0.746
BPH	2 (1.4)	17 (1.3)	11 (8.0)	30 (1.9)	<0.001
Chronic medication history					
ACEI	5 (3.6)	51 (3.8)	12 (8.8)	68 (4.2)	0.023
ARB	19 (13.7)	149 (11.2)	18 (13.1)	186 (11.6)	0.576
NSAID	10 (7.2)	82 (6.2)	20 (14.6)	112 (7.0)	0.017
Vaccination status					
No vaccination	137 (98.6)	1123 (84.4)	40 (29.2)	1300 (80.9)	<0.001
First dose completed	2 (1.4)	207 (15.6)	97 (70.8)	306 (19.1)	<0.001
Second dose completed	0 (0)	126 (9.5)	97 (70.8)	223 (13.9)	<0.001
Third dose completed	0 (0)	6 (0.5)	33 (24.1)	39 (2.4)	<0.001
Multiple admission					
Re-admitted cases	9 (6.5)	52 (3.9)	6 (4.4)	67 (4.2)	0.352

Table 3 reveals the clinical signs and symptoms of COVID-19 patients in three waves. Fever, cough, and headache were the most presenting symptoms in three waves. Clinical presentation with sore throat and rhinorrhea was observed more among the patients in the fourth wave of COVID-19 infection compared with previous waves.

**Table 3:** Clinical presentations of the COVID-19 patients on admission. COVID-19: coronavirus disease 2019

Clinical presentations	Second wave (n=139)	Third wave (n=1330)	Fourth wave (n=137)	Total (n=1606)	P-value (χ^2 ^test)
Fever	111 (79.9)	1076 (80.9)	95 (69.3)	1282 (79.8)	0.006
Cough	98 (70.5)	1008 (75.8)	105 (76.6)	1211 (75.4)	0.364
Shortness of breath	85 (61.2)	683 (51.4)	53 (38.7)	821 (51.1)	<0.001
Cough with sputum	56 (40.3)	547 (41.1)	63 (46.0)	666 (41.5)	<0.523
Myalgia	23 (16.5)	456 (34.3)	41 (29.9)	520 (32.4)	<0.001
Malaise	36 (25.9)	336 (25.3)	37 (27.0)	409 (25.5)	0.899
Loss of appetite	18 (12.9)	318 (23.9)	47 (34.3)	383 (23.8)	<0.001
Diarrhea	20 (14.4)	333 (25.0)	13 (61.2)	366 (22.8)	<0.001
Anosmia	10 (7.2)	225 (16.9)	3 (2.2)	238 (14.8)	<0.001
Sore throat	8 (5.8)	186 (14.0)	32 (23.4)	226 (14.1)	<0.001
Headache	7 (5.0)	169 (12.7)	13 (9.5)	189 (11.8)	0.019
Rhinorrhea	6 (4.3)	105 (7.9)	23 (16.8)	134 (8.3)	<0.001
Nausea/vomiting	10 (7.2)	62 (4.7)	17 (12.4)	89 (5.5)	0.001
Arthralgia	5 (3.6)	74 (5.6)	4 (2.9)	83 (5.2)	0.281
Inability to walk	8 (5.8)	46 (3.5)	13 (9.5)	67 (4.2)	0.002
Ageusia	0 (0)	59 (4.4)	0 (0)	59 (3.7)	0.002
Abdominal pain	5 (3.6)	35 (2.6)	8 (5.8)	48 (3.0)	0.101
Chest pain	8 (5.8)	31 (2.3)	6 (4.4)	45 (2.8)	0.034
Cough with hemoptysis	0 (0)	41 (3.1)	2 (1.5)	43 (2.7)	0.066
Altered consciousness	3 (2.2)	23 (1.7)	8 (5.8)	34 (2.1)	0.006
Hemorrhage	2 (1.4)	16 (1.2)	5 (3.6)	23 (1.4)	0.072
Insomnia	2 (1.4)	15 (1.1)	4 (2.9)	21 (1.3)	0.211
Wheezing	6 (4.3)	9 (0.7)	5 (3.6)	20 (1.2)	<0.001
Nasal stuffiness	2 (1.4)	8 (0.6)	4 (2.9)	14 (0.9)	0.016
Skin rash	0 (0)	6 (0.5)	1 (0.7)	7 (0.4)	0.641
Conjunctivitis	0 (0)	3 (0.2)	2 (1.5)	5 (0.3)	0.037
Seizures	0 (0)	4 (0.3)	0 (0)	4 (0.2)	0.660
Syncope	0 (0)	3 (0.2)	0 (0)	3 (0.2)	0.732
Skin ulcers	1 (0.7)	1 (0.1)	0 (0)	2 (0.1)	0.112
Lower chest wall indrawing	2 (1.4)	0 (0)	0 (0)	2 (0.1)	<0.001

**Figure 2:**
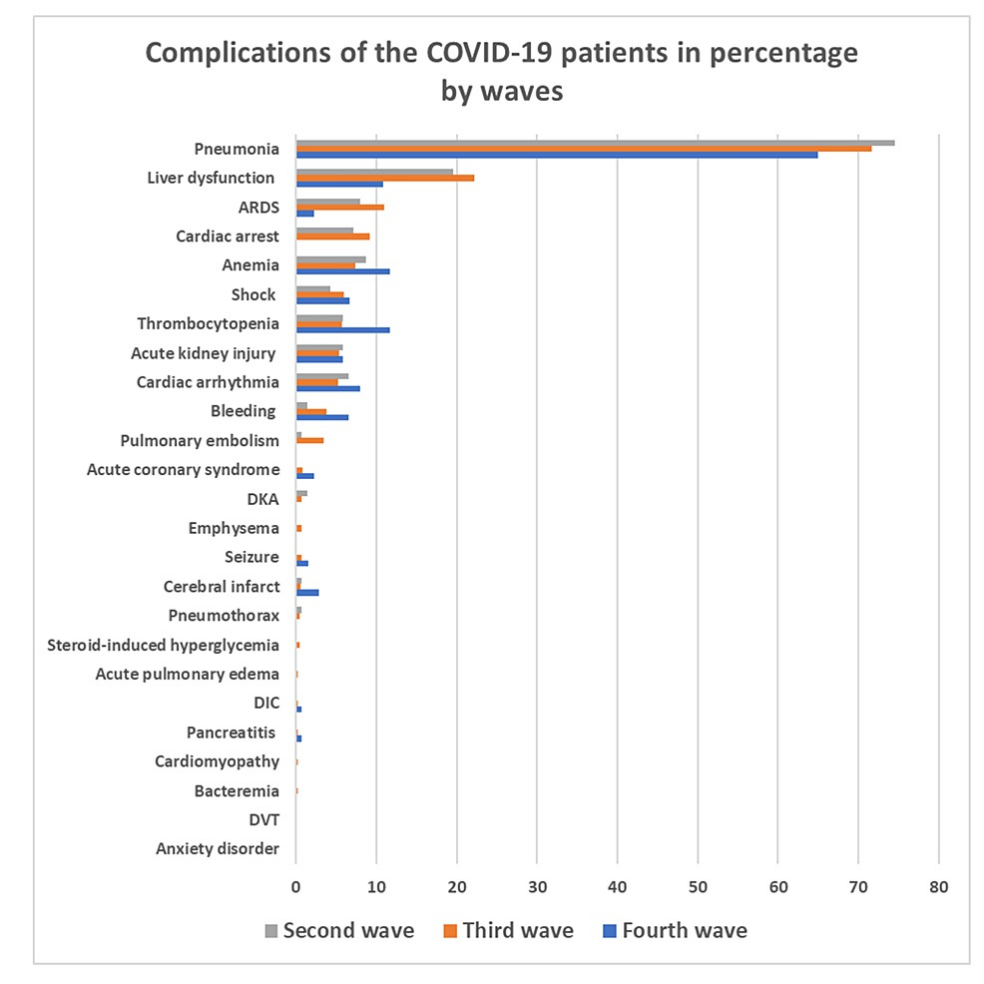
Complications of the COVID-19 patients in percentage by waves. ARDS, acute respiratory distress syndrome; DKA, diabetic ketoacidosis; DVT, deep vein thrombosis; DIC, disseminated intravascular coagulation; COVID-19, coronavirus disease 2019

The distribution of the complications of COVID-19 patients in three waves are shown in Figure 2. Among all patients, as a cumulative incidence during three waves, complication with pneumonia (71.3%), liver dysfunction (21.0%), ARDS (10.0%), thrombocytopenia (6.2%), acute kidney injury (5.5%), bleeding (3.9%), and pulmonary embolism (2.9%) were investigated. Respiratory complications such as ARDS and pneumonia were observed more in the second and third waves compared to the fourth wave. Liver dysfunction was also recorded less in the fourth wave compared to previous waves.

Table 4 shows the laboratory findings on admission, mid-time, and discharge in three waves of the COVID-19 patients. The reduction of mean lymphocyte count was observed among the patients during three waves especially in the second wave. Similarly, the increased median value of procalcitonin, hyper-sensitive C-reactive protein (hsCRP), and D-dimer were also recorded throughout three waves especially in second and third waves. High levels of the mean of ferritin were also reviewed among the patients in three waves especially in third and fourth.

**Table 4:** Laboratory findings of the COVID-19 patients during hospitalization by waves. Adm, on admission; Mid, midpoint of hospitalization; DC, discharge; WBC, white blood cell, INR, international normalized ratio; ALT, alanine transaminase; AST, aspartate transferase; hsCRP, hyper-sensitive C-reactive protein; LDH, lactate dehydrogenase; NA, not available; SD, standard deviation; COVID-19, coronavirus disease 2019; IQR, interquartile range

Variables		COVID-19 waves (mean±SD), median (IQR)			Total	
		Second wave (n=139)	Third wave (n=1330)	Fourth wave (n=137)		P-value
Hemoglobin, g/dL (mean±SD)	Adm	12.85±1.94 (n=139)	12.76±2.03 (n=1251)	12.39±2.47 (n=135)	12.73±2.07 (n=1525)	0.122
	Mid	12.76±2.32 (n=71)	12.45±1.99 (n=877)	12.07±1.98 (n=955)	12.44±2.02 (n=1043)	0.086
	DC	12.67±1.75 (n=106)	12.70±1.91 (n=1017)	12.33±2.33 (n=119)	12.67±1.94 (n=1242)	0.141
WBC count, 10^3^/µL (mean±SD)	Adm	8.69±4.44 (n=139)	8.49±5.54 (n=1251)	9.28±7.05 (n=135)	8.58±5.60 (n=1525)	0.294
	Mid	12.78±5.53 (n=71)	11.86±8.00 (n=876)	8.83±4.36 (n=95)	11.64±7.65 (n=1042)	<0.001
	DC	11.83±6.36 (n=106)	12.10±7.15 (n=1015)	9.42±5.11 (n=119)	11.82±6.95 (n=1240)	<0.001
Lymphocyte count, 10^3^/µL (mean±SD)	Adm	1.32±0.75 (n=138)	1.27±0.74 (n=1243)	1.29±0.77 (n=135)	1.27±0.74 (n=1516)	0.765
	Mid	1.29±1.22 (n=71)	1.42±0.95 (n=877)	1.68±0.88 (n=95)	1.43±0.97 (n=1043)	0.018
	DC	1.82±1.17 (n=106)	1.90±1.12 (n=1014)	1.96±1.18 (n=119)	1.90±1.13 (n=1239)	0.688
Hematocrit, % (mean±SD)	Adm	37.57±5.64 (n=138)	37.82±5.64 (n=1221)	36.95±8.48 (n=132)	37.72±5.95 (n=1491)	0.265
	Mid	37.45±6.49 (n=71)	37.06±5.65 (n=878)	36.64±5.98 (n=95)	36.96±5.75 (n=1044)	0.055
	DC	37.35±5.08 (n=106)	37.72±5.43 (n=1014)	35.98±6.41 (n=119)	37.52±5.53 (n=1239)	0.005
Platelet count, 10^3^/µL (mean±SD)	Adm	247.81±87.26 (n=139)	236.69±96.18 (n=1250)	236.86±96.99 (n=136)	237.72±95.47 (n=1525)	0.426
	Mid	317.49±131.05 (n=71)	268.25±119.91 (n=877)	237.88±80.19 (n=95)	268.84±118.62 (n=1043)	<0.001
	DC	346.36±330.71 (n=106)	297.23±123.74 (n=1016)	261.69±96.39 (n=119)	298.02±151.68 (n=1241)	<0.001
INR, median (IQR)	Adm	1.72±0.68 (n=12)	1.62±0.84 (n=62)	1.78±1.93 (n=16)	1.66±1.08 (n=90)	0.853
	Mid	NA	2.52±1.94 (n=13)	1.47±0.66 (n=2)	2.37±1.84 (n=15)	0.476
	DC	1.39 (n=1)	1.96±1.29 (n=13)	1.21±0.78 (n=2)	1.83±1.19 (n=16)	0.687
ALT, U/L, median (IQR)	Adm	26 (17.25-44) (n=128)	28 (17-47) (n=1193)	18 (13.5-27.5) (n=133)	26 (17-45) (n=1454)	<0.001
	Mid	44 (25.25-76.5) (n=60)	33 (21-56) (n=751)	19 (13-25) (n=87)	31 (20-54.25) (n=898)	<0.001
	DC	37 (24-59.5) (n=77)	35 (22-55) (n=835)	22 (14-31) (n=108)	33 (21-53.75) (n=1020)	<0.001
AST, U/L, median (IQR)	Adm	35.5 (25-54.5) (n=125)	34 (23-53) (n=1189)	26 (19-38) (n=134)	33 (23-51) (n=1448)	<0.001
	Mid	37 (27.25-53) (n=60)	31 (22-48) (n=748)	26 (21-33) (n=87)	30 (22-46) (n=895)	<0.001
	DC	26 (19-40) (n=76)	26 (19-38) (n=826)	24 (17.2-32.5) (n=108)	26 (19-37) (n=1010)	0.171
Total bilirubin, mg/dL, median (IQR)	Adm	0.5 (0.4-0.8) (n=127)	0.5 (0.3-0.7) (n=1192)	0.5 (0.3-0.7) (n=134)	0.5 (0.3-0.7) (n=1453)	0.007
	Mid	0.5 (0.4-0.67) (n=60)	0.5 (0.3-0.7) (n=749)	0.4 (0.3-0.6) (n=87)	0.5 (0.3-0.7) (n=896)	0.035
	DC	0.5 (0.4-0.8) (n=78)	0.5 (0.4-0.7) (n=835)	0.5 (0.3-0.7) (n=108)	0.5 (0.4-0.7) (n=1021)	0.380
Urea, mg/dL (mean±SD)	Adm	44.95±37.91 (n=106)	45.90±47.70 (n=509)	45.25±32.21 (n=42)	45.71±45.35 (n=657)	0.979
	Mid	75.56±73.37 (n=27)	70.03±58.25 (n=58)	NA	71.78±63.05 (n=85)	0.709
	DC	53.89±35.84 (n=48)	56.68±40.37 (n=120)	41.0±18.19 (n=3)	55.62±38.79 (n=171)	0.739
Creatinine, mg/dL, median (IQR)	Adm	1.0 (0.8-1.2) (n=136)	0.9 (0.8-1.2) (n=1225)	1.0 (0.8-1.3) (n=135)	0.9 (0.8-1.2) (n=1496)	0.053
	Mid	1.0 (0.8-1.3) (n=73)	0.9 (0.7-1.1) (n=813)	0.9 (0.7-1.2) (n=91)	0.9 (0.7-1.1) (n=977)	0.003
	DC	0.9 (0.7-1.1) (n=94)	0.8 (0.7-1.0) (n=938)	0.8 (0.7-1.1) (n=114)	0.8 (0.7-1.0) (n=1496)	0.012
Sodium, mmol/L (mean±SD)	Adm	136.35±5.25 (n=135)	136.36±4.52 (n=1221)	135.2±5.86 (n=134)	136.26±4.73 (n=1490)	0.025
	Mid	136.03±5.50 (n=71)	137.08±4.69 (n=818)	136.93±4.45 (n=96)	136.99±4.73 (n=985)	0.197
	DC	137.5±3.35 (n=93)	137.47±4.14 (n=919)	137.15±3.81 (n=113)	137.45±4.05 (n=1125)	0.726
Potassium, mmol/L (mean±SD)	Adm	4.03±0.47 (n=135)	4.03±0.61 (n=1221)	4.03±0.63 (n=134)	4.02±0.60 (n=1490)	0.962
	Mid	4.37±0.57 (n=70)	4.17±0.65 (n=819)	4.01±0.63 (n=96)	4.17±0.64 (n=985)	0.002
	DC	4.19±0.53 (n=93)	4.16±0.57 (n=917)	4.06±0.60 (n=113)	4.16±0.57 (n=1123)	0.177
Procalcitonin, ng/mL, median (IQR)	Adm	0.24 (0.18-0.32) (n=93)	0.12 (0.06-0.26) (n=373)	0.20 (0.11-0.45) (n=22)	0.17 (0.7-0.28) (n=488)	<0.001
	Mid	0.34 (0.22-0.40) (n=67)	0.21 (0.08-0.41) (n=195)	0.39 (0.21-6.45) (n=5)	0.27 (0.11-0.41) (n=267)	0.001
	DC	0.30 (0.22-0.37) (n=83)	0.10 (0.04-0.30) (n=249)	0.15 (0.06-0.33) (n=14)	0.19 (0.6-0.33) (n=346)	<0.001
hsCRP, mg/L, median (IQR)	Adm	61.1 (13.57-189.62) (n=138)	27.3 (7.57-105.9) (n=1242)	13.6 (4.5-39.5) (n=136)	26.57 (7.35-105.46) (n=1516)	<0.001
	Mid	44.6 (12.11-108.47) (n=68)	15.3 (6.3-38.6) (n=832)	15.6 (4.5-42.4) (n=89)	15.93 (6.3-40.79) (n=989)	0.010
	DC	5.22 (2.38-13.13) (n=101)	4.3 (1.48-12.15) (n=997)	4.5 (1.8-13.1) (n=116)	4.55 (1.57-12.21) (n=1214)	0.140
LDH, U/L (mean±SD)	Adm	341.29±173.0 (n=37)	372.87±205.12 (n=157)	463.4±626.11 (n=5)	369.28±216.55 (n=199)	0.450
	Mid	421.83±333.37 (n=6)	437.38±233.31 (n=32)	NA	434.92±246.29 (n=38)	0.889
	DC	315.42±212.09 (n=12)	308.92±157.78 (n=52)	257.0±130.09 (n=3)	307.76±165.45 (n=67)	0.860
D-dimer, µg/mL, median (IQR)	Adm	0.39 (0.20-0.96) (n=122)	0.41 (0.22-0.88) (n=1185)	0.49 (0.21-1.17) (n=133)	0.41 (0.22-0.93) (n=1440)	0.554
	Mid	1.02 (0.56-6.90) (n=51)	0.63 (0.30-1.64) (n=812)	0.44 (0.21-0.91) (n=86)	0.63 (0.30-1.62) (n=949)	0.004
	DC	0.77 (0.41-2.02) (n=85)	0.44 (0.25-1.01) (n=967)	0.42 (0.19-0.89) (n=112)	0.46 (0.25-1.05) (n=1164)	<0.001
Ferritin, ng/mL (mean±SD)	Adm	407.6±378.95 (n=6)	710.6±547.795 (n=248)	878.05±813.41 (n=11)	710.7±558.38 (n=265)	0.253
	Mid	366.5±308.9 (n=4)	859.95±521.38 (n=50)	1581 (n=1)	837.17±528.3 (n=55)	0.070
	DC	462.13±472.63 (n=4)	777.24±564.82 (n=86)	962.22±765.94 (n=6)	775.68±574.21 (n=96)	0.405

Treatment with intravenous fluids, oral fluids, corticosteroid, antiviral such as remdesivir, antibiotics such as fluoroquinolone, cephalosporin, and carbapenem was mostly used for the COVID-19 patients admitted at the hospital in three waves of the study period. Antiviral treatment had been used since the second wave, and antiviral drug such as remdesivir or molnupiravir was used more in the patients of the third and fourth waves than those of the second wave. Most of the patients were supplied with pipeline oxygen therapy. Non-invasive ventilation, invasive ventilation, and inotropes were observed in serious cases. Renal replacement therapy was also done in 1.1% of the cases during three waves. Disease severity during three waves was cumulatively showed as mild, moderate, severe, and critical cases with 56 (3.5%), 344( 21.4%), 871 (54.2%), and 335 (20.9%), respectively. Critical cases were observed mostly in the second wave compared to later waves (Table 5).

**Table 5:** Treatment and severity of the COVID-19 patients admitted at City Hospital. IV, intravenous; JAK, Janus kinase inhibitor; IL-6, interleukin 6; NSAID, nonsteroidal anti-inflammatory drug; ICU, intensive care unit; HDU, high-dependency unit; HFNC, high-flow nasal cannula; NIV, non-invasive ventilation; RRT, renal replacement therapy; COVID-19, coronavirus disease 2019

Treatments		Second wave (n=139)	Third wave (n=1330)	Fourth wave (n=137)	Total (n=1606)	P-value (χ^2 ^test)
Oral/orogastric fluids		28 (20.3)	150 (11.3)	5 (3.6)	183 (11.4)	<0.001
IV fluids		89 (64.5)	967 (72.7)	111 (81.0)	1167 (72.7)	0.009
Corticosteroid		108 (78.3)	979 (73.6)	84 (61.3)	1171 (73.0)	0.003
IL-6 inhibitor		9 (6.5)	106 (8.0)	0 (0)	115 (7.2)	0.003
JAK inhibitor		0 (0)	105 (7.9)	15 (10.9)	120 (7.5)	0.001
Monoclonal antibodies		0 (0)	1 (0.1)	0 (0)	1 (0.1)	0.901
Antiviral: remdesivir		52 (37.4)	805 (60.5)	104 (75.9)	961 (59.8)	<0.001
Antiviral: molnupiravir		0 (0)	2 (0.2)	12 (8.8)	14 (0.9)	<0.001
Antiviral: favipiravir		0 (0)	13 (1.0)	0 (0)	13 (0.1)	0.257
NSAID		13 (8.1)	125 (9.4)	22 (16.1)	160 (10.0)	0.046
Antibiotics						
Macrolides		11 (7.9)	43 (3.2)	7 (5.1)	61 (3.8)	0.016
Fluoroquinolones		84 (60.4)	495 (37.2)	63 (46.0)	642 (40.0)	<0.001
Third/fourth-generation cephalosporin		57 (41.0)	645 (48.5)	53 (38.7)	755 (47.0)	0.030
Carbapenem		41 (29.5)	422 (31.7)	32 (23.4)	495 (30.8)	0.122
Amoxicillin-clavulanate		50 (36.0)	246 (18.5)	13 (9.5)	309 (19.2)	<0.001
Cefoperazone-sulbactam		6 (4.3)	125 (9.4)	10 (7.3)	141 (8.8)	0.107
Piperacillin-tazobactam		22 (15.8)	48 (3.6)	8 (5.8)	78 (4.9)	<0.001
Gentamycin/amikacin		0 (0)	12 (0.9)	0 (0)	12 (0.7)	0.285
Linezolid/tedizolid		9 (6.5)	32 (2.4)	0 (0)	41 (2.6)	0.002
Metronidazole/ornidazole		4 (2.9)	25 (1.9)	6 (4.4)	35 (2.2)	0.136
Clindamycin/lincosamide		3 (2.2)	45 (3.4)	1 (0.7)	49 (3.1)	0.186
Vancomycin/teicoplanin		2 (1.4)	4 (0.3)	0 (0)	6 (1.8)	0.085
Supportive care						
ICU/HDU admission		40 (28.8)	228 (17.1)	13 (9.5)	281 (17.5)	<0.001
Oxygen therapy		99 (71.2)	798 (60.0)	65 (47.4)	962 (59.9)	<0.001
Interface	Nasal prongs	31 (22.3)	296 (22.3)	39 (10.7)	366 (22.8)	<0.001
	Masks	15 (10.8)	91 (6.8)	5 (3.6)	111 (6.9)	
	HFNC	31 (22.3)	61 (4.6)	0 (0)	92 (5.7)	
	Masks with reservoir	16 (11.5)	204 (15.3)	17 (12.4)	237 (14.8)	
	NIV mask	6 (4.3)	145 (10.9)	4 (2.9)	155 (9.7)	
Oxygen flow	1-5 L/minute	31 (22.3)	309 (23.2)	39 (28.5)	379 (23.6)	<0.001
	6-10 L/minute	18 (12.9)	90 (6.8)	8 (5.8)	116 (7.2)	
	11-15 L/minute	13 (9.4)	78 (5.9)	6 (4.4)	97 (6.0)	
	>15 L/minute	37 (26.6)	322 (24.2)	12 (8.8)	371 (23.1)	
Source of oxygen	Piped	98 (70.5)	787 (59.2)	65 (47.4)	950 (59.2)	<0.009
	Cylinder	1 (0.7)	10 (0.8)	0 (0)	11 (0.7)	
	Concentrator	0 (0)	1 (0.1)	0 (0)	1 (0.1)	
NIV		6 (4.3)	141 (10.6)	4 (2.9)	151 (9.4)	0.001
Invasive ventilation		0 (0)	8 (0.6)	0 (0)	8 (0.5)	0.436
Inotropes/vasopressors		4 (2.9)	64 (4.8)	6 (4.4)	74 (4.6)	0.580
Prone position		43 (30.9)	490 (36.8)	37 (27.0)	570 (35.5)	0.036
RRT		1 (0.7)	14 (1.1)	3 (2.2)	18 (1.1)	0.434
Severity of the diseases	Mild	5 (3.6)	48 (3.6)	3 (2.2)	56 (3.5)	0.064
	Moderate	27 (19.4)	285 (21.4)	32 (23.4)	344 (21.4)	
	Severe	64 (46.0)	727 (54.7)	80 (58.4)	871 (54.2)	
	Critical	43 (30.9)	270 (20.3)	22 (16.1)	335 (20.9)	

Treatment outcomes of the COVID-19 patients admitted at the hospital were described in Table 6. The mean duration of hospital stay in days was 10.8±5.94, 10.6±6.11, and 7.3±2.88 in the second, third, and fourth waves, respectively. About 90% of the hospitalized COVID-19 patients were discharged alive, and only 7.9% and 9.4% died in the hospital in the second and third waves. No mortality was observed in the fourth wave. There were 67 cases re-admitted as post-COVID-19 complications. Among them, only 13 (19.4%) had been vaccinated, and four (6%) died with post-COVID-19 complications.

**Table 6:** Outcomes of the COVID-19 patients admitted at City Hospital. SD, standard deviation; COVID-19, coronavirus disease 2019

Outcomes	Second wave (n=139)	Third wave (n=1330)	Fourth wave (n=137)	Total (n=1606)	P-value
Hospital stay in days (mean±SD)	10.8±5.94	10.6±6.11	7.3±2.88	10.3±5.96	<0.001 (t test)
Discharged alive	123 (88.5)	1162 (87.4)	124 (90.5)	1409 (87.7)	<0.001 (χ^2 ^test)
Palliative discharge	5 (3.6)	37 (2.8)	10 (7.3)	52 (3.2)	
Transferred to other facilities	0 (0)	6 (0.5)	3 (2.2)	9 (0.6)	
Death	11 (7.9)	125 (9.4)	0 (0)	136 (8.5)	

## Discussion

At the beginning of the COVID-19 pandemic in 2020, transmission was controlled by the Department of Public Health, and serious cases were treated at public hospitals of the Department of Medical Services under national treatment guidelines developed by the Ministry of Health. Later, a shortage of human resources resulted because of both the pandemic severity and the unstable country situation. Moreover, a surge in infection also led to the need for support by private sector involvement. Therefore, private hospitals started to manage COVID-19 infections in early 2021. This study evaluated the management of hospitalized patients by a private hospital in three different waves, the second, third, and fourth waves, in Myanmar. This study describes the clinical profile and outcomes of COVID-19 patients admitted at a private hospital in Mandalay, Myanmar. According to the findings, the private hospital in Mandalay could tackle COVID-19 severe cases in line with national treatment guidelines since the second wave and could provide better management in the fourth wave.

The mean age of the patients in the third wave was younger than those admitted in the second wave. COVID-19 immunization in Myanmar started in January 2021 with old-age people and healthcare workers as the first priority. Because of the immunization schedule mentioned earlier, older people were prioritized, and younger people who were going out for work were infected more than older people. This is similar to the studies of other countries in Asia [10-12]. However, the mean age of the patients in the fourth wave was older than those admitted in the second and third waves. In the later wave, people became familiar with mild-to-moderate infection and took self-care at home, and old people with severe infection took hospital care at a private hospital. Another reason may be because old people suffered more severe effects of the omicron variant than young people. Therefore, old age with severe cases should be closely monitored at the hospital. This finding is consistent with other studies [13-16]. Infection among healthcare workers was involved at 8.0%. The proportion was higher in the second and third waves with 8.6% each. However, it was lower in the fourth wave with 1.2%. It might be due to the vaccination effect among healthcare workers in the fourth wave, and the finding is similar to a retrospective study in India [17].

Comorbid conditions with hypertension or diabetes diseases were most commonly observed in three waves of COVID-19 infection in City Hospital, Mandalay. These were recorded with 48.2%, 39.8%, and 54.0% for hypertension and 41.0%, 34.3%, and 39.4% for diabetes in the second, third, and fourth waves, respectively. Therefore, patients with comorbid conditions of hypertension or diabetes and who are old should be taken with close care at the hospital. Although fever, cough, and headache were the most presenting symptoms in all waves, influenza-like symptoms such as sore throat and rhinorrhea were observed in high proportion in the fourth wave compared to previous waves. These findings were in contrast with the findings of the studies in Qatar and Iran [10,18].

Complications such as pneumonia, liver dysfunction, anemia, ARDS, cardiac injury, coagulopathy, acute kidney injury, and shock were observed in three waves. Respiratory complications such as ARDS and pneumonia were observed more in the second and third waves compared to fourth wave. Those are similar with a study in Wuhan and Qatar [14,18]. This led to higher proportion of oxygen therapy and higher proportion of ICU admission in the second and third waves compared to the fourth wave. This finding is also similar with the study done in 41 hospitals in India [19].

In this study, increased level of procalcitonin, C-reactive protein, and D-dimer were recorded among the patients throughout three waves especially in the second and third waves. Therefore, oxygen requirement with assisted ventilation was high among the patients who had high CRP and D-dimer in the second and third waves like other studies [10,20].

The hospital could manage the severe cases according to national treatment guidelines in all waves. Antiviral treatment was observed more in patients of the third and fourth wave than those of the second wave because of the less proportion of severe cases needed for antiviral treatment in the second wave, or locally recommended antiviral treatment were not available in the area or hospital in that period. Nevertheless, antiviral treatment might lead to better outcomes in the fourth wave. Similarly, treatment with non-invasive ventilation and invasive ventilation was used in more patients of the third wave compared to those of other waves. These findings are similar to those found in Africa [21].

The mean duration of hospital stay was shortened in the fourth wave compared to previous waves. This finding is also consistent with other studies [18,21]. No mortality was observed in the fourth wave. This successful result may be reflected from experienced practices of the hospital achieved in previous waves. Therefore, it showed that hospital management became better in the fourth wave compared to previous waves [22,23].

Limitations of the study

This study is a retrospective record review, so it is not a prospective study. This study describes the clinical profile of COVID-19 patients by assessment with CRF developed using the WHO Global COVID-19 Clinical Data Platform. The CRF contains three modules: module 1, to be completed on the first day of admission to the health center; module 2, to be completed daily during hospital stay for as many days as resources allow and to continue to follow-up patients who transfer between wards; and module 3, to be completed at discharge or death. However, the current study reviewed the patients’ records using WHO module that was modified. It could not be completed during hospital stay and continued to follow-up. The record review was done after the patients were discharged. Therefore, the assessment might not be complete enough. Another limitation is that although there were four waves in Myanmar, the clinical profile of the patients of the first wave was not studied in this research. In this connection, the emergency response and control activities were sufficient with available and efficient workforce in 2020. Therefore, private hospital involvement was not needed in 2020, resulting in no studies relating private hospital management in the first wave of 2020. In addition, laboratory results were reviewed on available findings in EMR. Many laboratory investigations of the patients were not available, and those were not included in the analysis. Moreover, clinical outcomes of the admitted patients were determined by reviewing the EMR, and those who were discharged or referred to other facilities were not followed. This study was conducted in one private hospital only; it was not done as a multicenter study.

## Conclusions

At the beginning of the COVID-19 pandemic in 2020, cases were contained and treated at public hospitals in Myanmar. However, because of an unstable country situation, the private hospitals supported the management of the COVID-19 crisis in the subsequent waves: Delta in 2021 and Omicron in 2022. This study recommended that COVID-19 patients with comorbid conditions of hypertension or diabetes and who are 65 years and older should be taken with intensive care support at the hospital and should be encouraged for vaccination. This study also concluded that a private hospital in Mandalay could tackle COVID-19 severe cases in line with national treatment guidelines since the second wave and could provide better management in the fourth wave. Antiviral treatment should be used in severe COVID-19 cases for further emergency management. In conclusion, private hospital involvement in the COVID-19 pandemic is supportive of the healthcare provision in Myanmar in an emergency situation.
